# Does Language Matter? Identity-First Versus Person-First Language Use in Autism Research: A Response to Vivanti

**DOI:** 10.1007/s10803-020-04858-w

**Published:** 2021-01-20

**Authors:** Monique Botha, Jacqueline Hanlon, Gemma Louise Williams

**Affiliations:** 1 School of Psychology, University of Surrey, Stag Hill Campus, Guildford, Surrey GU2 7XH UK; 2 LLM Gender, Conflict & Human Rights, Transitional Justice Institute, Ulster University, Jordanstown, UK; 3 School of Humanities, University of Brighton, Brighton, Sussex BN2 4AT UK; 4 Present Address: Division of Psychology, Faculty of Natural Sciences, University of Stirling, Stirling, UK

**Keywords:** Person-first language, Identity-first language, Stigma, Rights-based approach, Autism

## Abstract

In response to Vivanti’s ‘Ask The Editor…’ paper [*Journal of Autism and Developmental Disorders, 50*(2), 691–693], we argue that the use of language in autism research has material consequences for autistic people including stigmatisation, dehumanisation, and violence. Further, that the debate in the use of person-first language versus identity-first language should centre first and foremost on the needs, autonomy, and rights of autistic people, so in to preserve their rights to self-determination. Lastly, we provide directions for future research.

## Introduction

In this article, we expand on the conversation regarding the use of person first language (PFL), and identity first language (IFL), by responding to the editorial by Vivanti (2020). Vivanti attempts to answer the question “what is the most appropriate way to talk about individual with a diagnosis of autism?” (Vivanti 2020). We begin by reiterating the core arguments around PFL and IFL as highlighted by Vivanti, and by reiterating the strengths of the article—such as its focus on the material implications of language for autistic people. We then expand further on the implications of linguistic framings which separate autistic people from their autism—namely violence. We highlight how the article might have been strengthened with further engagement with the growing body of critical autism research. We further expand on the rights-based approach to language Vivanti briefly discusses, highlighting considerations for communication adjustments, the externalisation of vulnerability, and that self-determination should be the centre of any discussion of disability. Lastly, we discuss the direction of future empirical work which might address any perceived ambiguity around the language verbal, nor non-speaking autistic people prefer.^1^ In doing so, we highlight that autistic people (across all ranges of speaking, and with or without learning disabilities) should be directing the language used to describe us. It should also be noted that speaking and non-speaking is not dichotomous because autistic people can move between speaking in some environments or spaces and not in others, and this is a dynamic and not static group (Peña 2019). Importantly, non-speaking does not mean non communicative, or non-thinking (Peña 2019).

In his ‘Ask the Editor’ piece, Vivanti (2020) provides a brief history of the language used to describe disabilities, including the tensions between IFL (e.g. “autistic person”) and PFL (e.g. “person with autism”). The author details how PFL (which emerged in the 1970s) was used to put the person before the disability—to emphasise “the person’s unique combinations of strengths, needs, and experiences (both related and unrelated to their disability)… by literally placing the person before the disability” (Vivanti 2020: 1). IFL, the author argues, “is increasingly endorsed as an expression of positive social identity whereby language historically used to dehumanize and marginalise… is redeployed as a form of empowerment” (Vivanti 2020: 2).

We would first like to commend Vivanti for the positive contributions of his editorial. The very fact that the Journal of Autism and Developmental Disorders is addressing this sometimes-perplexing issue is most welcome. Above all, we were especially glad to see Vivanti recognise the real-world implications of language choice: how it can ‘influence societal perceptions, public policy, clinical practice and research directions’ (Vivanti 2020: 1). Later, too, he reminds readers that all disabled people—including autistic people—are vulnerable to experiencing violations of their human rights and highlights how non-speaking autistic people may have additional difficulties in advocating for their rights, and may therefore be ‘more at risk to be treated as “less than people”’ (Vivanti 2020: 2). It is both right and important that these issues are considered by researchers working in the field of autism research and the journals who publish them.

## Finding Consensus

We would like to acknowledge, early on, that currently there is no clear consensus in *literature*, regarding the most preferred language from autistic people—as alluded to by Vivanti (2020). However there is, we believe, clear consensus on the least preferred—and most offensive—language: the specific person-first formulation of “person with autism” or “person with autism spectrum disorder/condition” (Bury et al. 2020; Kapp et al. 2013; Kenny et al. 2016). Perhaps confusingly, while “autistic” and “autistic person” remain the preferred term by largest agreement (totalling 49% in a recent study by Bury et al. 2020), “on the autism spectrum” has shown to be the least polarizing overall (ibid.).

A person “on the spectrum” is essentially, a person first formulation and yet is found to be substantially less offensive than “with autism” by the autistic community (Bury et al. 2020; Kapp et al. 2013; Kenny et al. 2016; Sinclair 2013). This poses a conundrum, and potentially lends support to the argument that a move completely away from PFL may be premature (Bury et al. 2020; Vivanti 2020). However, linguistic analysis may help to explain the different connotations that these two formulations (“person with autism” and “person on the autism spectrum”) carry.

The first case (“person with autism”) involves the noun (“person”) followed by “with + noun” (here: “autism”). Other nouns (or noun phrases) that may be substituted in the second position for “autism” include those such as “cancer”, “liver disease”, “a headache”, “Covid-19”, etc. This is common formulation used to signify the presence of some kind of pathology or illness. In the second case (“person on the spectrum/autism spectrum”) represents a far less common formula, and as such already carries fewer connotations. However, the “…on the spectrum” can be substituted with prepositional phrases such as “on the cricket team”, “on the engineering course”, or “on holiday”, etc. These substitutions all denote position of some sort and provide a means of both locating the subject (the “person”) and identifying them as a member of a smaller group of people (i.e. the members of the category “person” is far greater than those belonging to the category of “person on the forecourt”).

We agree with Bottema-Beutel et al. (2020: 3), that ‘what people say or write produces specific versions of the world, one’s self, and others, and language conveys, shapes, and perpetuates ideologies’. We would therefore also like to lend our support to their suggestion that “person on the autism spectrum” be used as a less offending middle ground between two more politically entrenched ways of talking about autism only *where* such a compromise is necessitated. In doing so, we reject the assertion by Vivanti that it is too early to reject PFL, if he means the continued use of “person with autism” because a not insubstantial body of literature is showing that this is both least preferred and most offensive, and there are more preferred, and less offensive alternatives available.

## The Material Risk of Language for Autistic People

Autism is both a “real” phenomenon, and constituted from social meaning, culture, language, and common understanding (Davidson and Orsini 2013; Nadesan 2013; Yergeau 2018). This means that language shapes our understanding of autism, and can have risks, as acknowledged by Vivanti (2020). Real world risks to autistic people are clear, as evidenced by the heightened incidence rates of self-harm, post-traumatic stress disorder, suicidal ideation and death by suicide among autistic people, compared to a non-autistic population (Cassidy et al. 2014; Cassidy and Rodgers 2017; Haruvi-Lamdan et al. 2020; Hedley et al. 2018; Hirvikoski et al. 2016), and the high rate of interpersonal victimisation (Griffiths et al. 2019; Weiss and Fardella 2018). Terminology around autism and, specifically, the language used to refer to autistic people may have a significant role to play in those risks, as language frames concepts, thought, perception and stigma (Gernsbacher 2017). Indeed, there has been a history both within academia (Gernsbacher 2007; Cowen 2009) and in news reporting of autism (Holton et al. 2014; Huws and Jones 2011) of using dehumanising and stigmatising language. PFL was originally designed in the 1970s as a response to the dehumanisation and violence towards autistic and disabled people of earlier decades. The thinking of the time was that it may humanise disabled people in a way IFL did not, in the face of institutional violence against disabled people because it is a formulation which puts the person first (Dunn and Andrews 2015), yet emerging evidence has not found it to be efficacious (Gomes 2018). The changes in formulation, historically, have not prevented violence against autistic people which is still occurring despite moving between formulations. It is important to note that the original use of IFL was not used to reflect the preference of autistic people but rather reflected the medical language of the time, whereas the current IFL movement reflect the ever-growing push from autistic and disabled people for autonomy (Botha et al. 2020; Dunn and Andrews 2015). Further, PFL may have unintended consequences which we discuss. We argue that this editorial has not gone quite far enough in analysing the potentially damaging implications of at least, some formulations of person-first language.

Linguistic framing influences the way that people conceptualise social matters, political attitudes, events, and situations (Bergen 2012; Reali et al. 2016). Metaphors are one of the linguistic tools used to frame, for example mental illness, as an opponent (Reali et al. 2016). Further, they do not serve as ornamental features of language, and instead play an active role in conceptualising reality (Reali et al. 2016). In fact, when considering how people frame depression for example, Reali et al. (2016) found that mere exposure to linguistic cues is enough to produce effects on first impression, biasing participants towards making certain interpretations over other available interpretations. There might be other consequences for these types of framings—most importantly, violence against autistic people. Campaigns and language around autism have had “problematic discursive figuration of autism as somehow separate from the autistic person” (McGuire 2016: 160), something PFL serves to solidify. Person-first language literally serves to drive a wedge between the person (good), and the autism (bad) (McGuire 2016).

Consider the New York University Child Study Center’s ‘public service campaign’ consisting of ‘ransom notes posted on large billboards, on kiosks, and at construction sites in New York City and published in *Newsweek* and *New York* magazine’ (Kras 2010: n.p.), written from the perspective of ‘autism’ that had kidnapped innocent children. Autism here was positioned outside of the child them self, as a malevolent force that would ‘destroy’ their life- a common narrative in autism (McGuire 2016). Similar themes can be found in several publicity videos for the charity Autism Speaks. In one, entitled “I am Autism”, ‘a satanic-sounding voice declares “I am autism” and proceeds to brag about all the destruction he will cause families’ (Nicolaidis 2012: 504). More consideration needs to be given to what metaphors, framings and ways of talking about autism the linguistic separation creates. If autism can be separated from the person (a goal of PFL), then metaphors around the destruction of it may be used without consideration for the life attached—autism becomes an opponent, a disembodied force. In fact, the United States enacted an autism bill named the “Combating Autism Act”, which many saw as congress “declaring war on autism” (McGuire 2016). We are reminded of the apt quote by Anne McGuire “When autism is not a life but a shell surrounding life, advocacy is positioned to see normal kids, as per the opening epigraph, that simply need to be ‘cracked’ out” (2016). Often, it is forgotten autism *only* exists as a person.

The implications of the semantic separation of autism and people are most clear in cases of filicide. Autism is a risk factor for ‘altruistic’ filicide (ASAN 2020; Palermo 2003), and in a review of 26 filicide-suicides reported in newspapers between 1982 and 2010 in the USA, 54% of victims were autistic (Coorg and Tournay 2013), despite making up between only 1 and 2% of the population (Baron-Cohen et al. 2009). In the past five years ‘over 650 people with disabilities have been murdered by their parents, relatives or caregivers’ (ASAN 2020: 4). These, of course, are just the reported cases. Of the five possible categorisations for the murder of a child by their parents, ‘altruistic filicide’ is the by far the most common (Palermo 2003; Sobsey 2001), and ‘in cases that involve disabled persons, the concept of altruistic filicide may dangerously overlap the idea of mercy killing’ (Palermo 2003: 47). McGuire (2016) highlights how in cases of filicide, parents often fall back on the same linguistic separation—I loved my child very much, but I hated autism, and wanted autism out of my life. More disturbing, was an ideology that by killing an autistic child, the child would somehow be complete (non-autistic) in heaven (McGuire 2016). This is clear in testimony on the killing of Katie McCarron, in which the mother claims she was not killing her autistic daughter, but rather autism itself (Sampier 2008). Conceptual (and linguistic) separation of the individual and ‘their autism’ hurts all those on the spectrum, perhaps especially those who are multiply disabled and, as Vivanti pointed out, often even less able to assert their human rights independently. By separating autism from the person, we formulate an existence or possibility of that life without autism (McGuire 2016), yet autism cannot, ontically speaking, ever be separate from a person: to combat autism, is to combat autistic people. While historically PFL was intended to humanise and acknowledge the individuality of each person, there remain concerns about the impact of such semantic separations of people from their disabilities (Dunn and Andrews 2015).

## Superficial Engagement with Autistic Scholarship

Whilst we recognise that Vivanti’s (2020) original editorial was not designed to be a comprehensive examination of the literature, we would like to note one serious limitation in its lack of engagement with the literature produced by autistic scholars addressing these issues (such as Arnold 2017; Chapman 2019, 2020; Chown 2019; Graby 2015; Milton in Milton and Timimi 2016; Woods and Waldock 2020; Sinclair 2013; Singer 1999, 2017; Walker 2012). Our response, in part, attempts to add to that body of (already substantial) work. We were gratified to note Vivanti’s mention of the disability rights movement when providing background for the use of person-first language: bringing this to the attention of the journal’s readership is commendable. However, for an article that, on the surface, appears to argue for the importance of remembering that autistic people ‘are people first’ (Vivanti 2020: 1), it seems a shame that the central tenet of disability rights activity—‘*nothing about us without us*’ (Charlton 2004)—has been overlooked in practice within the editorial’s discussion.

It is now increasingly recognised that autism research both greatly benefits from autistic input, and is simultaneously, sorely lacking in it (Chown et al. 2017; Gillespie-Lynch et al. 2017; Gowen et al. 2019; Happé and Frith 2020; Milton 2014; Milton and Bracher 2013; Nicolaidis 2012; Pellicano 2014; Pellicano et al. 2014; Pellicano and Stears 2011; Woods and Waltz 2019). Yet this is not for a lack of autistic scholars or self-advocates working in a participatory capacity alongside non-autistic researchers. There is a lack of nuance, in Vivanti’s (2020) editorial, around the representation of the (so-called) neurodiversity movement’s conceptualisation of autism that would have benefitted from stronger engagement with autistic voices. Vivanti (2020: 1) claims, for example, that the ‘neurodiversity perspective… sees autism as an expression of cultural diversity, rather than pathology’. This is simply not true. From a neurodiversity perspective, autism is not a cultural phenomenon , it is a biological one. The concept of neurodiversity simply highlights that there is s dispersion of cognitive and biological function in humans. While some have embraced an autistic cultural identity, ‘neurodiversity’ more accurately refers to biological (in this case, neurological) human diversity (Singer 1999, 2017; Walker 2012). Likewise, whilst the neurodiversity perspective does not view autism as pathological (as Vivanti 2020, rightly asserted), it is in full recognition of the many ways in which autistic people can be disabled, it just does not ascribe disability solely within the individual: an important factor that is lost when autism or neurodiversity is spoken about as a simply being cultural diversity (Nadesan 2013).

Again, when returning to the discussion of appropriate language, Vivanti (2020: 1) explains that:

Identity-first language (e.g., autistic person, blind person) is considered as an appropriate expression of this cultural shift [to a neurodiversity perspective] by many self-advocates and scholars, as it counteracts the risk that separating the individual from the diagnosis (as in the expression “person with autism”) perpetuates the societal view that something is wrong about the diagnosis, potentially leading to an internalization of inferiority for those who receive the diagnosis.

Whilst we applaud the underscoring of the risks attached to person-first language, we feel a significant detail has been misunderstood. A neurodiversity approach proposes not a ‘cultural shift’ but a *paradigmatic* one (Chapman 2019; Walker 2012): away from one that pathologises neurological difference. Person-first language belongs to the pathology paradigm and, as such, ‘implicitly accepts and reinforces the assumption that Autism is intrinsically a problem, a Something-Wrong-With-You’ (Walker 2012: n.p.). It affects how physicians ‘think of our patients’ and implicitly encourages ‘family members to love an imagined non-autistic child that was never born, forgetting about the real person who exists in front of us’ (Nicolaidis 2012: 505).

## Human Rights and Vulnerability

Vivanti also addresses human rights, correctly emphasising that each autistic person, no matter the severity of their impairment, is entitled to respect for their human rights. The United Nations Convention on the Rights of Persons with Disabilities (CRPD 2008) however, goes beyond *respect* of disabled person’s human rights; it requires that disabled people are guaranteed the full *enjoyment* of their human rights *without distinction* (CRPD 2008: preamble, our emphasis). This challenges traditional, paternalistic approaches to disability which portrays impairment as an inherent vulnerability (Celik 2017). Article 8 requires that states combat stereotypes, such as the assumption made here that non-speaking adults are ‘less equipped to advocate for themselves’ (Vivanti 2020: 693), while article 5 requires reasonable accommodation to ensure non-discrimination in the exercise of convention rights. For non-speaking adults, this includes the recognition and use of alternative modes of communication to facilitate self-advocacy (Nicolaidis et al. 2015).

The European Court of Human Rights (ECtHR) has similarly moved towards a legal understanding of vulnerability that moves away from it being an inherent condition (Arnardóttir 2017). In *Alajos Kiss v Hungary*, for example, the court recognises that ‘such groups were historically subject to prejudice with lasting consequences, resulting in their social exclusion’ (*Alajos Kiss v Hungary* 2010: 42). That is, the basis of harm is the exclusion of groups of people based on pervasive and harmful stereotypes (Arnardóttir 2017). Further, the ECtHR follows the CRPD by placing a positive obligation on the state to remove barriers to participation through reasonable accommodation (*Guberina v Croatia* 2016). To ensure the inclusion of a broader range of autistic people in research, this may include co-creation of survey instruments to ensure accessibility for the widest range of participants, using graphics and simple language, and offering definitions and clarification of more complex terminology (Nicolaidis et al. 2020).

A human rights-based approach must not reinforce systems which create vulnerability, including systems of language which perpetrate stereotypes and, by implication, categorise people as vulnerable. Although Vivanti avoids the use of the terms high and low functioning, the use of other epithets such as ‘most impaired end of the spectrum’ and ‘minimally verbal individuals’(Vivanti: 692) still implies vulnerability is linked to specific functional markers rather than social context. The term ‘minimally verbal’ is, itself, one that has a basis in linguistics research, such as that of Tager-Flusberg and Kasari (2013). However, whilst the term is meant as descriptive, large inconsistencies exist in how it is applied, and according to which linguistic and paralinguistic criteria: perhaps in part due to the heterogeneity of the non-speaking autistic population (Koegel et al. 2020). We feel that sweeping uses of such unclear terms as this, which carry negative connotations ought to be avoided so as not to endorse or perpetuate stereotypes because they may become a euphemism for ‘low functioning’. Further, non-speaking does not tend to mean non- or minimally-verbal because non-speaking autistic people still tend to communicate in less socially valued ways.

The harm caused by stereotypes is addressed in Kenny (2016) and is an area where there is clear agreement between the respondent groups in the survey. When discussing the high and low functioning autism label, all groups indicated that they view these terms as a misleading and value-based judgement on a person’s ability to function (Kenny et al. 2016). Assumptions based on these labels minimise the difficulties of those who appear high functioning and underestimate the ability of those viewed as low functioning (Kenny et al. 2016). One of the family and friends group makes specific reference of non-verbal communication, stating that they find ‘the idea of language being a definer of ‘function’ out-dated and frankly quite arrogant’ (Kenny et al. 2016: 449). There is a significant risk that ‘minimally verbal’ and ‘most impaired end of spectrum’ become replacement catch-all terms for the stereotyping autistic people are actively trying to escape in eschewing functional labels. It is vital, then, to challenge approaches which may, albeit unintentionally, perpetrate negative stereotypes.

We welcome Vivanti’s concern that tensions over language may contribute to the privileging of one group within the autism community over another (692), but do not share agreement with his solutions. Researchers, practitioners, and clinicians discounting narratives of minorities in favour privileging their own perspectives is a danger often seen in minority-based work (McKinnon 2017) and disability research (Wieseler 2020) and it constitutes a type of violence. Often autistic people who can and do engage with researchers are dismissed for not being autistic enough, while those who use few to no words are systematically kept out of research. Vivanti (2020) perpetuates this by arguing that the autistic people included in the samples of current research may have different perspectives on language because they are seen as being *more* able (which is conflated with being *less* autistic). Instead of dismissing current testimony, we believe that the lack of representative evidence that involves a full spectrum of autistic people should be addressed by research approaches which are participatory or consensus building (Nicolaidis et al. 2015; Bertilsdotter Rosqvist et al. 2020).

The participatory approach used by Nicolaidis et al. (2015) indicates that it is possible to create inclusive research and that the research can show commonality of experience across the spectrum of communication difficulties. For example, lack of reasonable accommodation to communication styles impacts on autonomy (Nicolaidis et al. 2015: 828) among both respondents using assistive communication technology and those who have large vocabularies, who both felt they were condescended to and infantilised (828). Improved outcomes were created by the person providing care implementing reasonable adjustments, effective communication and respecting autonomy of the person (829). Similarly, Bertilsdotter Rosqvist et al. (2020) highlight the possibilities of creating ‘autistic shared spaces’ (Bertilsdotter Rosqvist et al. 2020: 168). Moving the focus of concern away from hierarchies and harmful divisions within the autism community (Hillary 2020) towards enabling equal participation of respondent groups that can help move away from stereotyping autistic people as fixed within their own ‘bubbles’ (Bertilsdotter Rosqvist et al. 2020: 169). Allowing research to be guided by disabled people in a manner which respects inherent dignity of all disabled persons and allows them the freedom to make their own choices (CRPD: article 3) is more consistent with a human-rights based approach.

## Directions for Future Research

A common limitation of surveys include difficulty in reaching the entire autism spectrum, as highlighted in the studies that address preferred language used to describe autism (Bury et al. 2020; Kenny et al. 2016). Reaching autistic populations at intersections with learning disabilities and situational mutism can be difficult, and the methods usually used are restrictive, and potentially exclude a representative sample. The sample methods used can often result in samples who use a wider vocabulary, were diagnosed later in life, and have higher educational attainment. However, there is often also a troubling assumption that anyone who participates in surveys are typically verbal, and that those who are situationally mute, or non-speaking would not have taken part. Vivanti (2020) refers to ‘minimally verbal’ autistic people but does not clarify whether they are referring to those with co-occurring learning disabilities, situational mutism, perceived developmental language delays, or a constellation of these characteristics. This casual conflation usually spans from the assumption that non-speaking autistic people, or those who are situationally mute always have a co-occurring learning disability, and cannot communicate in any method, because they do not *always* communicate verbally. Further, researchers often categorise a heterogenous group of autistic people together based on the perception of one function-normative verbal communication. Yet 75% of autistic children with co-occurring situational mutism do not have learning disabilities (Steffenburg et al. 2018), while in the most complete census of any country to date, 20% of autistic people have a co-occurring learning disability (Kinnear et al. 2019). There are intersections of these populations who might not use traditional communication, or use few to no words, and yet this nuance is rarely acknowledged. Not all people who do not use spoken language use no communication methods at all. It fails to acknowledge that non-speaking autistic adults are a similarly heterogenous group deserving of nuanced discussion. In future, rather than stereotyping a group of autistic people by a specific single, normative skill, perhaps researchers should point to a specific constellation of characteristics they are referring to.

To date, research has not collected information on this as there is an assumption if one takes part in a survey then they are automatically conventionally verbal, and further that they always have been. It should be noted that in a qualitative study that included adjustments to allow for the participation of those who do not communicate verbally, but may communicate through typing for example, there was still a preference for IFL (Botha et al. 2020). Ultimately, there needs to be more distinction when referring to autistic people, less assumptions on behalf of researchers, and indeed, greater nuance and further research.

Further, there is a problematic divide issued on the basis of conventional verbal ability: Vivanti (2020) argues that PFL is purposed to counteract judgements made of these groups especially (those who are non-speaking) (Vivanti 2020: 2). Firstly, autistic people are dehumanised by non-autistic people, regardless of communication type (Cage et al. 2018). Secondly, there is no evidence that PFL effectively humanises disabled people (Collier 2012). But most importantly, no autistic individual, regardless of how they communicate, should be *so* dehumanised that they require language emphasising their humanness. If autistic people who are verbal do not need PFL, but non-speaking autistic people need it, is it because it is somehow harder to remember that non-speaking autistic individuals are human? As a society, rather than having to remind people of the fundamental humanness of a marginalised minority, we should instead be tackling the preconceived notions of those who need the reminder, that this is in fact, a person in front of them—something autistic people, both verbal and non-speaking, have made clear (Botha et al. 2020).

Thus, with regards to concerns about sampling issues perhaps the path forward is to conduct a replication study of the regularly cited Kenny et al. (2016) paper on language preference, that includes expanded methods to reach a wider sample of the autism spectrum, using a participatory method that encompasses more of the autistic spectrum, and its intersectionality. This replication could additionally ask two things: (1) how verbal they would currently consider themselves, and (2) how verbal they were as a child (as those who communicate conventionally as adults might not have done so as children), and the co-occurrence of learning disabilities and situational mutism. This would remove the perceived ambiguity of language preference according to perceived verbalness.

Further research should also aim to understand identity first and PFL with more nuance—including *why* the different person first formulations ( “on the autism spectrum” is perceived as the least polarizing, while “on the autism” spectrum is routinely considered, with reasonable consensus, to be the most offensive). Research could focus on the linguistic nuance in interpretation and meaning making of these formulations. What is arguably most important in these directions for future research is that it remains person centred. That is, research and practice are framed around the needs, wishes, and experiences of autistic individuals most particularly. Directions in how we talk about autism in research ought to follow the guidance of autistic people themselves. In the meantime, while further research is needed, it should certainly be heeded that the autistic community does not tend to favour “with autism”.

## Conclusion

There will not always be consensus on what autism is or how we should talk about autistic people—even among autistic people ourselves—but to ignore those autistic voices that have broken through into the academy lest they not be representative of every single autistic person is short-sighted. Linguistic framing, including the use of PFL, has material consequences for the autistic community, especially those who are non-speaking. The priority of research should be to centre autistic people (both speaking and non-speaking, and with, and without learning disabilities) in the conversation around the language used to describe autism and autistic people. If current studies are not representative enough of the entire autistic population the solution is to conduct more research. We would urge Vivanti and contributors to the Journal of Autism and Developmental Disorders to engage more fully with critical autism studies and other autistic academic voices on all matters of autism research for the mutual benefit of the both the autistic and the research communities.

## Footnotes

1 We use ‘non-speaking’ in this paper where others, including Vivanti (2020) sometimes use ’non-verbal’ or ‘minimally verbal’, as this is anecdotally reported as being the preferred terminology by those to whom it refers (see: https://xminds.org/Nonspeaking-Autistics).
